# Identification of candidate aberrantly methylated and differentially expressed genes in Esophageal squamous cell carcinoma

**DOI:** 10.1038/s41598-020-66847-4

**Published:** 2020-06-16

**Authors:** Bao-Ai Han, Xiu-Ping Yang, Davood K Hosseini, Po Zhang, Ya Zhang, Jin-Tao Yu, Shan Chen, Fan Zhang, Tao Zhou, Hai-Ying Sun

**Affiliations:** 1 Public Laboratory, Key Laboratory of Breast Cancer Prevention and Therapy, Ministry of Education, Tianjin Medical University Cancer Institute and Hospital, National Clinical Research Center for Cancer, Tianjin Medical University, Tianjin, 30000 China; 2 Department of Otorhinolaryngology, Union Hospital, Tongji Medical College, Huazhong University of Science and Technology, Wuhan, 430022 China; 3 Department of Otolaryngology-Head and Neck Surgery, Stanford University School of Medicine, Stanford, 94305 United States; 4 Department of Otorhinolaryngology, Head and Neck Surgery, Zhongnan Hospital of Wuhan University, Wuhan, 430071 China; 5 Department of Medicine, Stanford University School of Medicine, Stanford, 94305 United States; 6 Department of Neurosurgery Tongji Hospital, Tongji Medical College, Huazhong University of Science and Technology, Wuhan, 430030 China; 7 Guangdong Provincial Maternal and Child Health Care Hospital, Guangzhou, 511400 China

**Keywords:** Cancer epidemiology, Cancer epigenetics

## Abstract

Aberrant methylated genes (DMGs) play an important role in the etiology and pathogenesis of esophageal squamous cell carcinoma (ESCC). In this study, we aimed to integrate three cohorts profile datasets to ascertain aberrant methylated-differentially expressed genes and pathways associated with ESCC by comprehensive bioinformatics analysis. We downloaded data of gene expression microarrays (GSE20347, GSE38129) and gene methylation microarrays (GSE52826) from the Gene Expression Omnibus (GEO) database. Aberrantly differentially expressed genes (DEGs) were obtained by GEO2R tool. The David database was then used to perform Gene ontology (GO) analysis and Kyoto Encyclopedia of Gene and Genome pathway enrichment analyses on selected genes. STRING and Cytoscape software were used to construct a protein-protein interaction (PPI) network, then the modules in the PPI networks were analyzed with MCODE and the hub genes chose from the PPI networks were verified by Oncomine and TCGA database. In total, 291 hypomethylation-high expression genes and 168 hypermethylation-low expression genes were identified at the screening step, and finally found six mostly changed hub genes including KIF14, CDK1, AURKA, LCN2, TGM1, and DSG1. Pathway analysis indicated that aberrantly methylated DEGs mainly associated with the P13K-AKT signaling, cAMP signaling and cell cycle process. After validation in multiple databases, most hub genes remained significant. Patients with high expression of AURKA were associated with shorter overall survival. To summarize, we have identified six feasible aberrant methylated-differentially expressed genes and pathways in ESCC by bioinformatics analysis, potentially providing valuable information for the molecular mechanisms of ESCC. Our data combined the analysis of gene expression profiling microarrays and gene methylation profiling microarrays, simultaneously, and in this way, it can shed a light for screening and diagnosis of ESCC in future.

## Introduction

Esophageal squamous cell carcinoma (ESCC) is one of the most common malignancies worldwide, with incidence rates ranking fourth and eighth in China and worldwide, respectively^1,2^. ESCC is a serious threat to human health, and its five-year survival rate is less than 10%, making it a leading cause of cancer-related death^3^. So far, the molecular mechanism of the occurrence and development of ESCC has not been fully clarified. Currently, one of the main directions of ESCC research is searching for key genes or specific biomarkers that affect the occurrence and development of ESCC to determine genetic susceptibility factors in ESCC and clarify their molecular mechanisms^4^. Therefore, the cloning and identification of new genes related to ESCC are of great significance in terms of early warning signs, early clinical diagnosis, treatment, prognosis monitoring and the development of new anticancer drugs for susceptible individuals.

Epigenetics refers to the heritable alteration of gene expression unrelated to changes in DNA sequence^5,6^. DNA methylation is the most common epigenetic change, and it is closely related to gene expression regulation^7^, embryonic development^8^, X chromosome inactivation^9^, genomic stability^10^ and genomic imprinting^11^, and plays a significant role in cancer progression. DNA methylation of gene promoters is associated with silencing of key genes, especially oncogenes and tumor suppressors and is considered to be a hallmark in many carcinomas^12^. Recently, some studies have confirmed that certain genes with abnormal DNA hypermethylation or hypomethylation are involved in various processes of ESCC development^13,14^, but it is still difficult to determine the comprehensive profile and pathways of the interaction network.

Comprehensive analysis of multiple datasets provides the capabilities to properly identify and assess the pathways and genes that mediate the biological processes associated with ESCC. In the present research, we used publicly available datasets of gene expression (GSE20347, GSE38129) and gene methylation (GSE52826) to identify genes and pathways that were both abnormally methylated and differentially expressed between tumor tissues and normal esophagus from patients with ESCC. The selected genes were then subjected to hierarchical clustering, and functional enrichment analyses of GO and KEGG pathways. Then we used STRING to create protein-protein interaction networks and identify hub genes that were key to these signaling events. Furthermore, we validated the results using the Cancer Genome Atlas (TCGA) data to identify DMGs involved in the pathogenesis of ESCC. Finally, the GEPIA database was utilized to perform survival analysis. We believe our data provide comprehensive biological information about novel differentially methylated genes and enhance the level of understanding regarding the development and progression of ESCC.

## Results

### Identification of abnormally methylated and differentially expressed genes in ESCC

**Figure 1:**
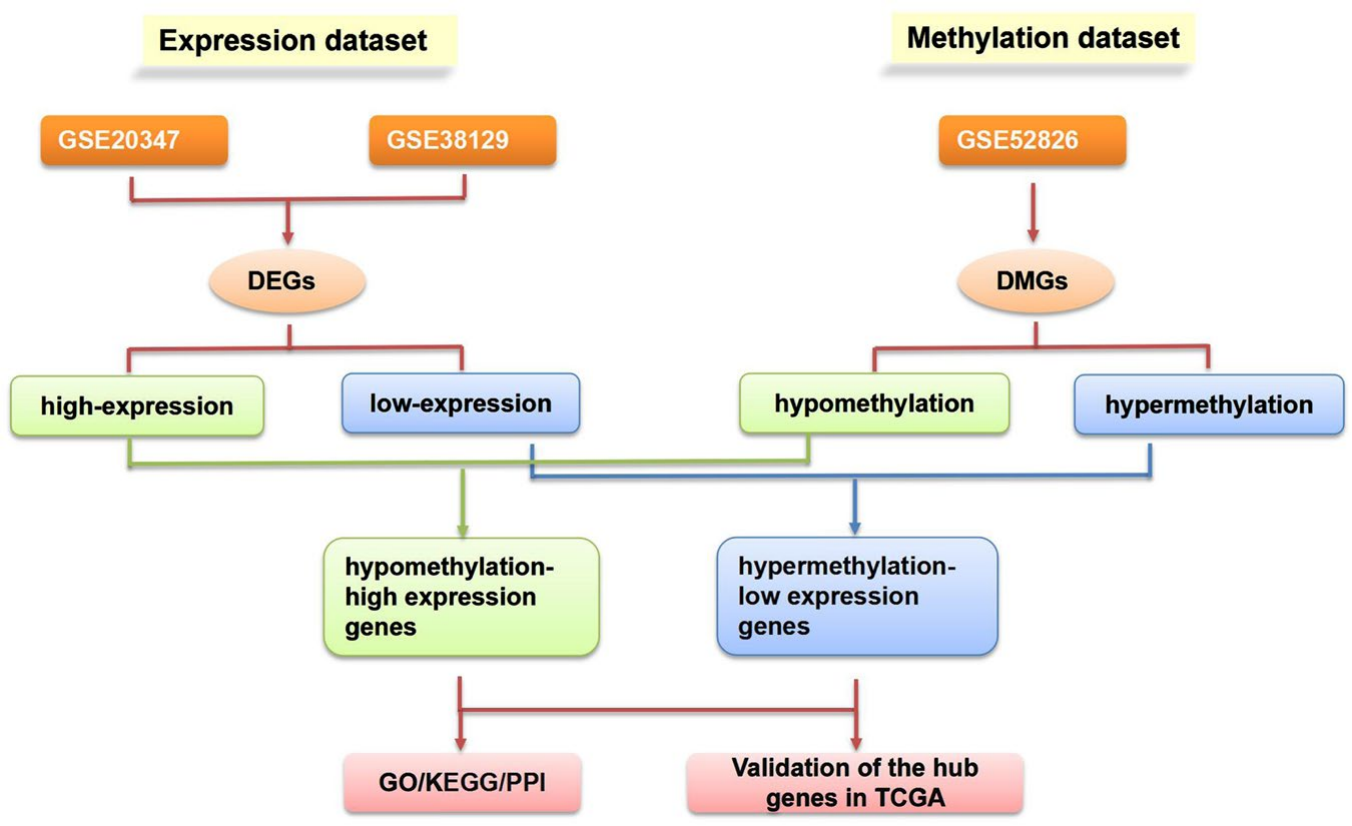
The flowchart of our study. DMG: differentially methylated gene; DEG: differentially expressed gene; GO: gene ontology; KEGG: Kyoto Encyclopedia of Genes and Genomes; PPI: protein-protein interaction; TCGA: The Cancer Genome Atlas.

**Figure 2:**
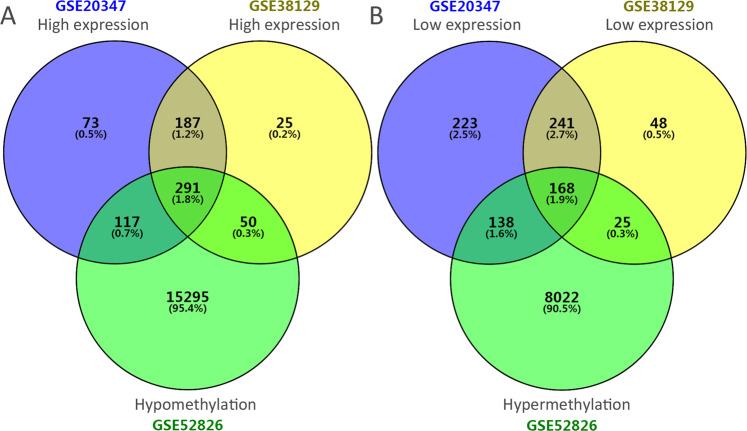
Identification of aberrantly methylated and differentially expressed genes was analyzed by Funrich software. Different color areas represented different datasets. (**A**) Hypomethylation and high expression genes; (**B**) hypermethylation and low expression genes.

**Figure 3:**
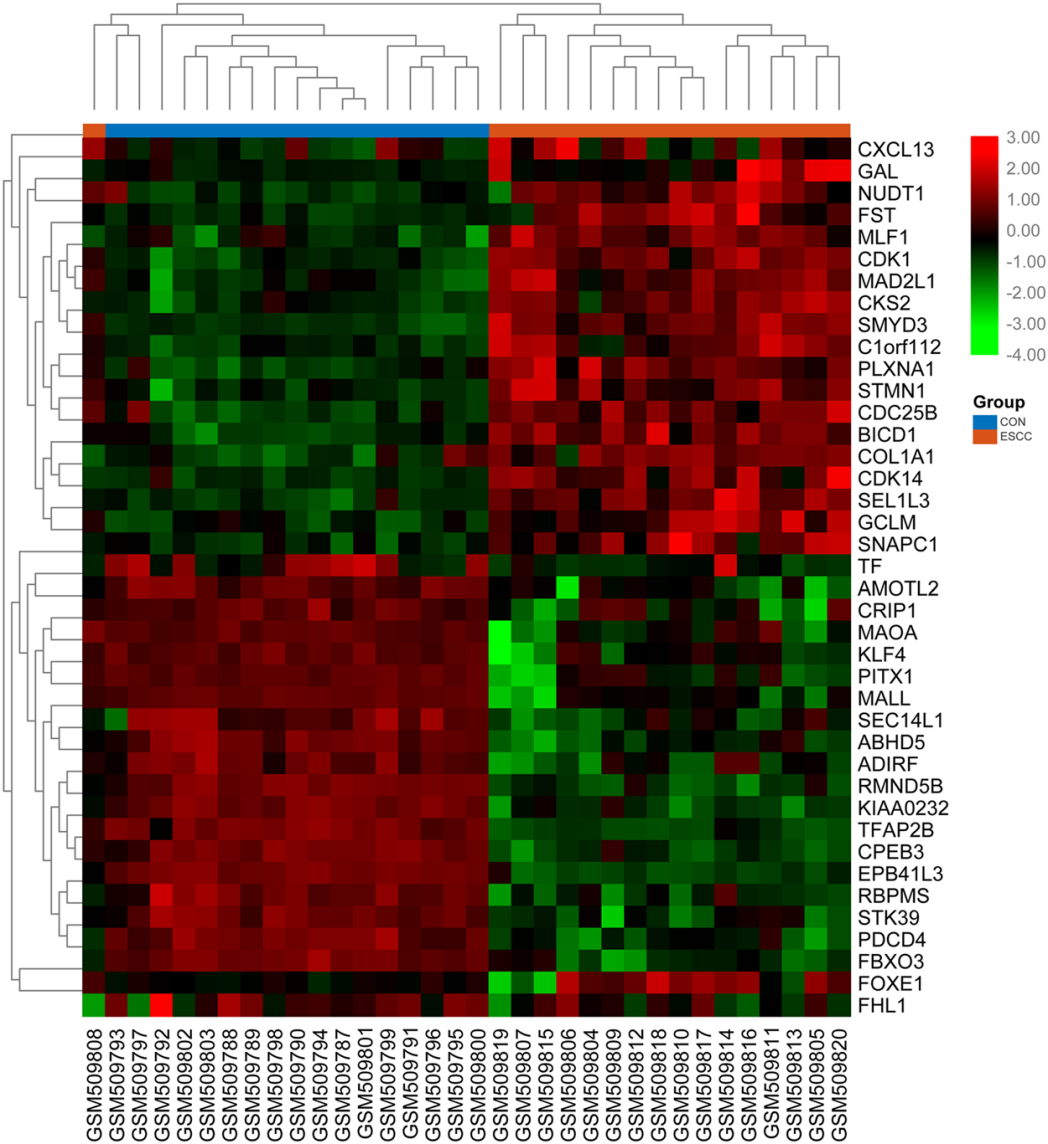
The heat map of top 20 hypomethylation/high-expression genes and top 20 hypermethylation/low-expression genes in GSE20347.

The flowchart of our study is demonstrated in Fig. 1. For differentially expressed genes (DEGs) of the gene expression microarray, 478 overlapping upregulated genes (668 in GSE20347 and 553 in GSE38129) and 409 overlapping downregulated genes (770 in GSE20347 and 482 in GSE38129) were identified. For DMGs of the gene methylation microarray, 8353 hypermethylated genes and 15753 hypomethylated genes were identified. Then, a total of 291 hypomethylated/upregulated genes were obtained by overlapping 15753 hypomethylated genes and 478 upregulated genes; 168 hypomethylated/downregulated genes were obtained by overlapping 8353 hypermethylated genes and 409 downregulated genes (Fig. 2). The heat map of the top 20 hypomethylated/upregulated genes and the top 20 hypomethylated/downregulated genes in GSE20347 is shown in Fig. 3.

### Gene ontology and pathway functional enrichment analysis

**Figure 4:**
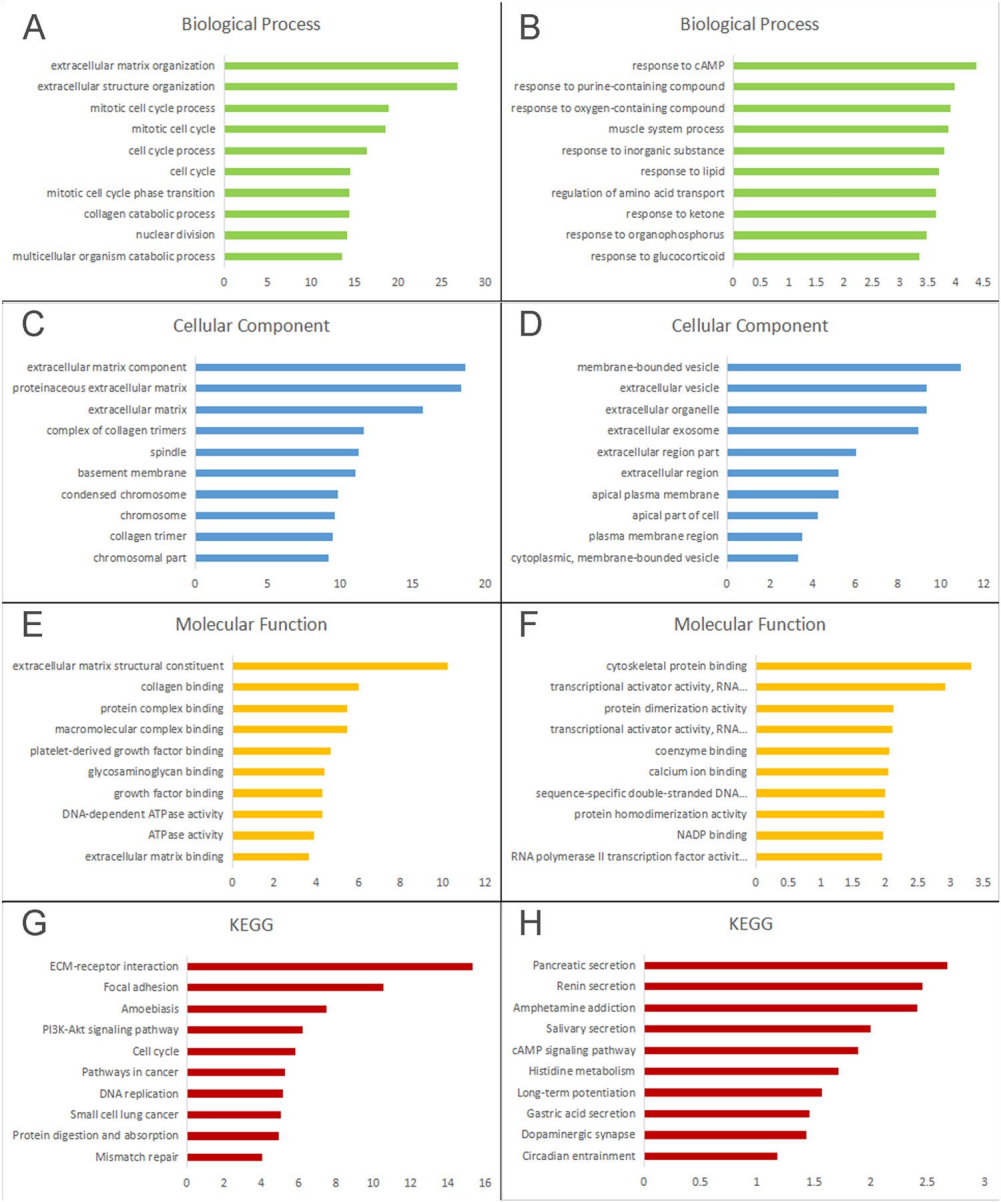
The gene ontology annotation and pathway enrichment analysis of all the aberrantly methylated and differentially expressed genes. (**A**) Biological process, (**C**) cellular component, (**E**) molecular function, and G: KEGG of hypomethylation/high-expression genes. (**B**) Biological process, (**D**) cellular component, (**F**) molecular function, and (**H**) KEGG of hypermethylation/low-expression genes. The high enrichment score means that the genes were found more frequently in the particular ontology. KEGG, Kyoto Encyclopedia of Genes and Genomes P value was shown in the Supplementary Material.

GO annotation and pathway enrichment analyses of the 291 mutually inclusive hypomethylated/high-expression and 168 hypermethylated/low-expression genes were implemented using the online tool DAVID. Genes that were hypomethylated and highly expressed were enriched in extracellular matrix organization, extracellular structure organization, mitotic cell cycle process, cell cycle process, mitotic cell cycle phase transition, collagen catabolic process, nuclear division and multicellular organism catabolic process (Fig. 4A), while the hypermethylated low-expression genes were primarily linked to the cell response to cAMP, purine-containing compound, oxygen-containing compound, inorganic substance, lipid, ketone, organophosphosphorus, glucocorticoid, molecules involved in regulation of amino acid transport, and muscle system process (Fig. 4B). Cell component enrichment analysis indicated that hypomethylated highly-expressed genes were correlated with extracellular matrix component, proteinaceous extracellular matrix, complex of collagen trimers, spindle, basement membrane, condensed chromosome, collagen trimer and chromosomal part (Fig. 4C), whereas hypermethylation/low-expression genes were predominantly enriched in membrane-bounded vesicle, extracellular vesicle, extracellular organelle, extracellular exosome, extracellular region part, apical plasma membrane, apical part of cell and plasma membrane region (Fig. 4D). For molecular function, hypomethylated/high-expression genes were enriched mainly in extracellular matrix structural constituent, collagen binding, protein complex binding, macromolecular complex binding, platelet-derived growth factor binding, glycosaminoglycan binding, growth factor binding, DNA-dependent ATPase activity, ATPase activity and extracellular matrix binding (Fig. 4E), while hypermethylated/low-expression genes were mostly enriched in cytoskeletal protein binding, transcriptional activator activity, RNA polymerase II transcription regulatory region sequence-specific binding, protein dimerization activity, coenzyme binding, calcium ion binding, sequence-specificdouble-stranded DNA binding, protein homodimerization activity and NADP binding (Fig. 4F). The pathway analysis revealed that hypomethylated highly-expressed genes were linked to ECM-receptor interaction, Focal adhesion, Amoebiasis, PI3K-Akt signaling pathway, Cell cycle, Pathways in cancer, DNA replication, Small cell lung cancer, Protein digestion and absorption and Mismatch repair (Fig. 4G), while hypermethylated/low-expression genes were significantly enriched in Pancreatic secretion, Renin secretion, Amphetamine addiction, Salivary secretion, cAMP signaling pathway, Histidine metabolism, Long-term potentiation, Gastric acid secretion, Dopaminergic synapse and Circadian entrainment (Fig. 4H). These screened pathways demonstrated that DEGS and DMGs have a crucial function in the tumor microenvironment, etiology and pathogenesis of ESCC.

### Construction and analysis of PPI networks

**Figure 5:**
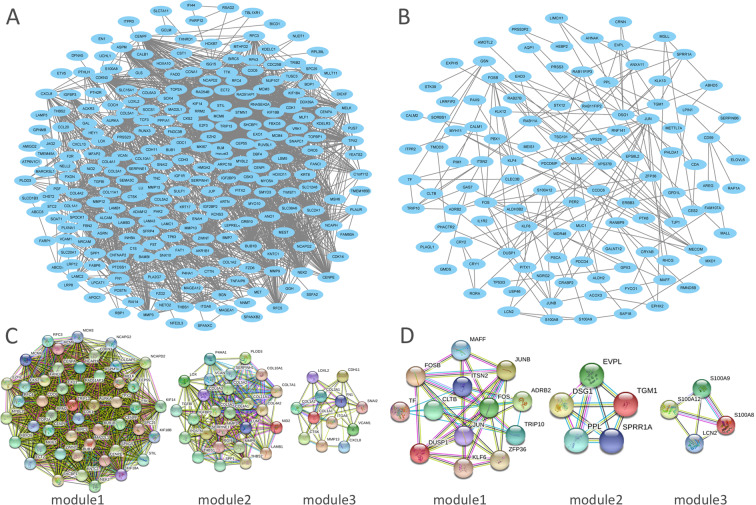
PPI network and Top 3 modules of aberrantly methylated and differentially expressed genes. (**A**) PPI network of hypomethylation/high-expression genes and (**B**) PPI network of hypermethylation/low-expression genes. (**C**) Top 3 modules of hypomethylation/high-expression genes and (**D**) Top 3 modules of hypomethylation/high-expression genes. PPI, protein-protein interaction.

**Figure 6:**
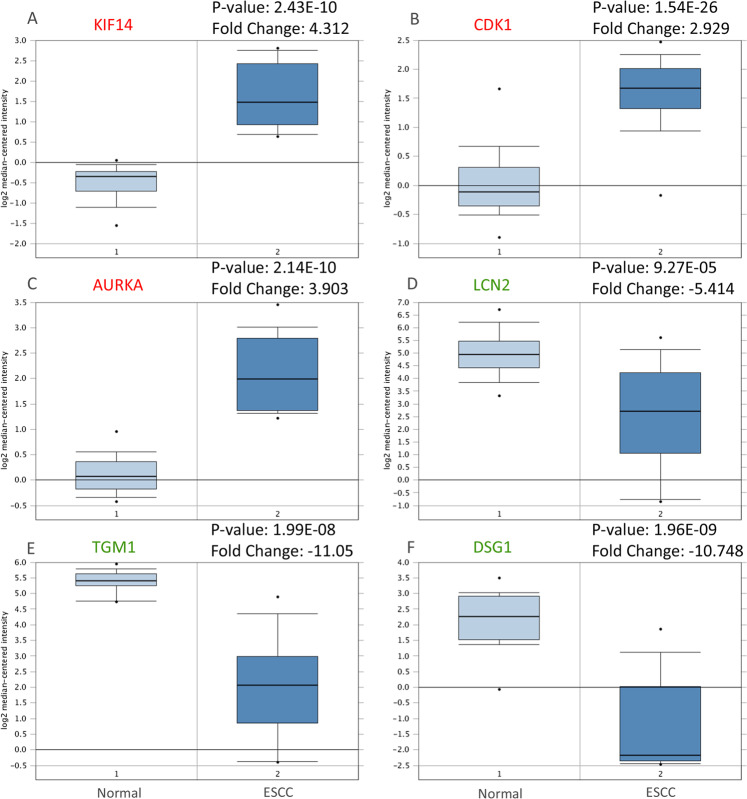
Validation of the expression of hub genes in Oncomine database. The expression level of **(A**) KIF14, **(B**) CDK1, **(C**) AURKA, **(D**) LCN2, **(E**) TGM1, and **(F**) DSG1 were detected in Oncomine database. Red: Hypomethylation/ high-expression genes; Green: Hypermethylation/low-expression genes.

The STRING database was applied for PPI network construction, with MCODE applied for module analysis. Hub genes were identified using the cytoHubbaCytoscape software. The PPI network for genes that were hypomethylated and highly expressed is demonstrated in Fig. 5A, with corresponding modules shown in Fig. 5C. The most significantly enriched functional modules were those linked to Cell cycle, DNA replication, Oocyte meiosis, Mismatch repair, Progesterone-mediated oocyte maturation, Nucleotide excision repair and p53 signaling pathway (Table 1). The top three hub genes were KIF14, CDK1 and AURKA. The PPI network for genes that were hypermethylated and expressed at low levels is demonstrated in Fig. 5B, with corresponding modules demonstrated in Fig. 5D. Significant modules showed functions including Osteoclast differentiation, Amphetamine addiction, IL-17 signalling pathway, TNF signalling pathway, Fluid shear stress and atherosclerosis, Kaposi’s sarcoma-associated herpes virus infection and cAMP signalling pathway (Table 1). The top three hub genes were LCN2, TGM1 and DSG1. Furthermore, we used the Oncomine database to confirm the expression of hub genes in ESCC (Fig. 6). The data are consistent with our outcomes.

**Table 1:** Module analysis of the protein–protein interaction network.

Category	Pathway description	FDR	Nodes	Genes
Hypomethylation and high expression				
Cell cycle		6.93E-10	9	BUB1, BUB1B, CDC6, CDK1, MAD2L1, MCM3, MCM4, MCM6, TTK
DNA replication		5.96E-07	5	MCM3, MCM4, MCM6, FRC3, FRC4
Oocyte meiosis		9.11E-05	5	AURKA, BUB1, CDK1, FBXO5, MAD2L1
Mismatch repair		2.10E-04	3	EXO1,RFC3, RFC4
Progesterone-mediated oocyte maturation		4.60E-04	4	AURKA, BUB1,CDK1, MAD2L1
Nucleotide excision repair		2.46E-02	2	RFC3, RFC4
p53 signaling pathway		4.37E-02	2	CDK1, RRM2
Hypermethylation and low expression				
Osteoclast differentiation		8.33E-05	4	FOS, FOSB, JUN, JUNB
Amphetamine addiction		3.90E-04	3	FOS, FOSB, JUN
IL-17 signaling pathway		7.10E-04	3	FOS, FOSB, JUN
TNF signaling pathway		8.50E-04	3	FOS, JUN, JUNB
Fluid shear stress and atherosclerosis		1.20E-03	3	DUSP1, FOS, JUN
Kaposi’s sarcoma-associated herpesvirus infection		2.60E-03	3	FOS, JUN, ZFP36
cAMP signaling pathway		2.70E-03	3	ADRB2, FOS, JUN

### Validation of the hub genes

**Figure 7:**
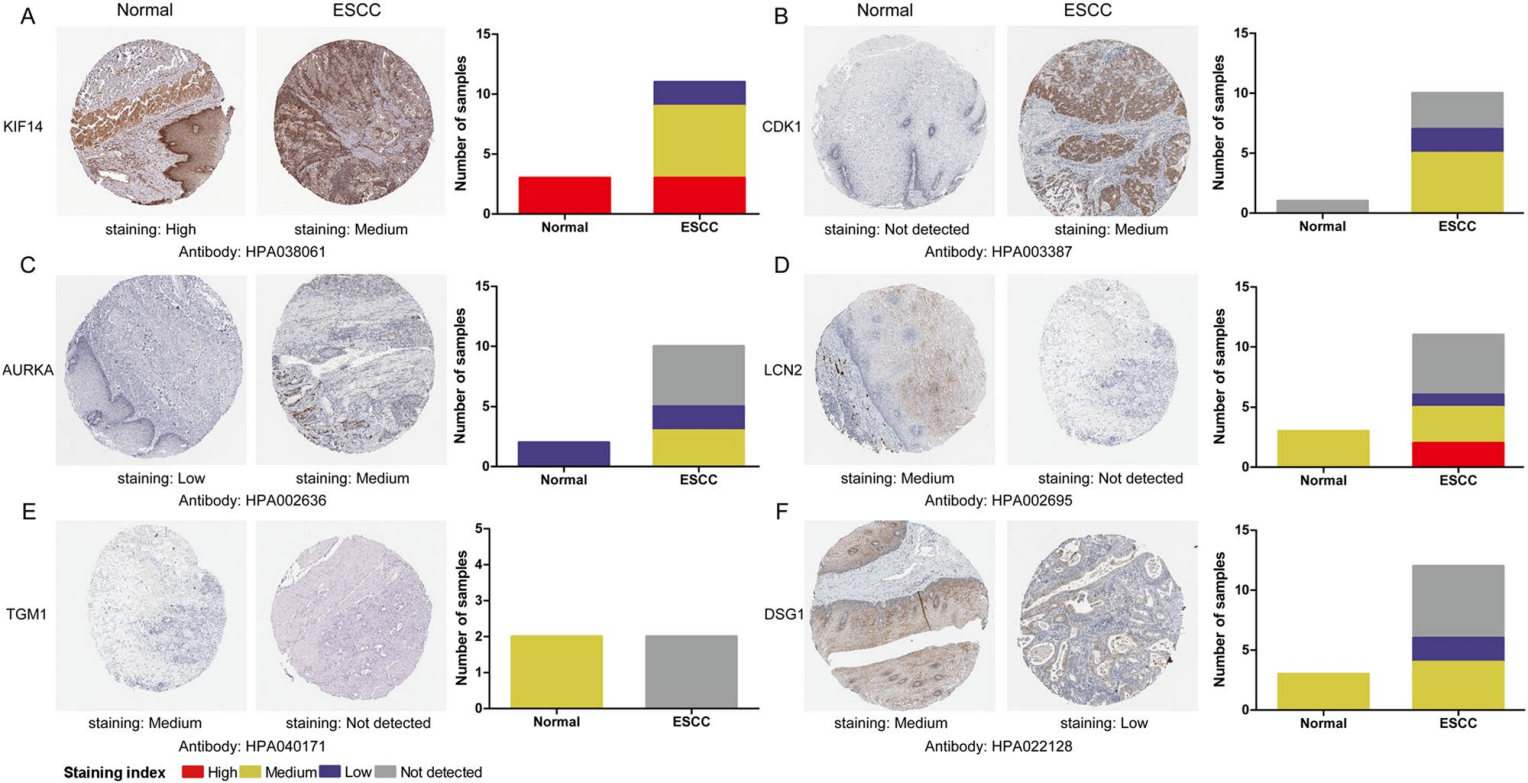
Validation the expression of the hub gene on translational level by the Human Protein Atlas database. (**A**) KIF14, (**B**) CDK1, (**C**) AURKA, (**D**) LCN2, (**E**) TGM1, (**F**) DSG1.The staining strengths were annotated as Not detected, Low, Medium and High. The bar plots indicating the number of samples with different staining strength in HPA database.

Hypermethylated/low-expression hub genes and hypomethylated/high-expression hub genes were then validated in another database TCGA to confirm the outcomes. The outcomes were summarized in Table 2. For most of the hub genes, the expression status and methylation were significant changed and similar to our outcomes, which indicates the reliability and stability of the findings. Then, we performed analysis of the protein expression patterns of the Hub gene in ESCC, by utilizing data available from the Human Protein Atlas (Fig. 7). The results showed that the KIF14 protein gene was highly expressed in normal esophageal tissues, whereas medium expression was observed in ESCC tissues. No expression of CDK1 was observed in normal tissues, while medium CDK1 gene expression was appreciated in tumor tissues. Low gene expression of AURKA was observed in normal tissues and medium expression was observed in ESCC tissues. LCN2 and TGM1 were found to have medium expression in normal esophageal tissues, while no expression was observed in ESCC tissues. In addition, medium expression of DSG1 was observed in normal tissues, while low expression of DSG1 was observed in tumor tissues. This result is consistent with most of our previous observations.

**Table 2:** Validation of the hub genes in TCGA database.

Hub gene	Methylation status	P value	Expression status	P value
**Hypomethylation/high-expression**				
KIF14	Hypomethylation	2.800E-02	High expression	5.447E-19
CDK1	Hypomethylation	1.170E-07	High expression	1.41E-18
AURKA	Hypomethylation	4.572E-08	High expression	2.30E-15
**Hypermethylation/low-expression**				
LCN2	Hypermethylation	0.024	Low expression	0.018
TGM1	Hypermethylation	1.121E-04	Low expression	0.069
DSG1	Hypermethylation	1.400E-02	Low expression	0.02

### The relationship between hub genes and the survival in ESCC

**Figure 8:**
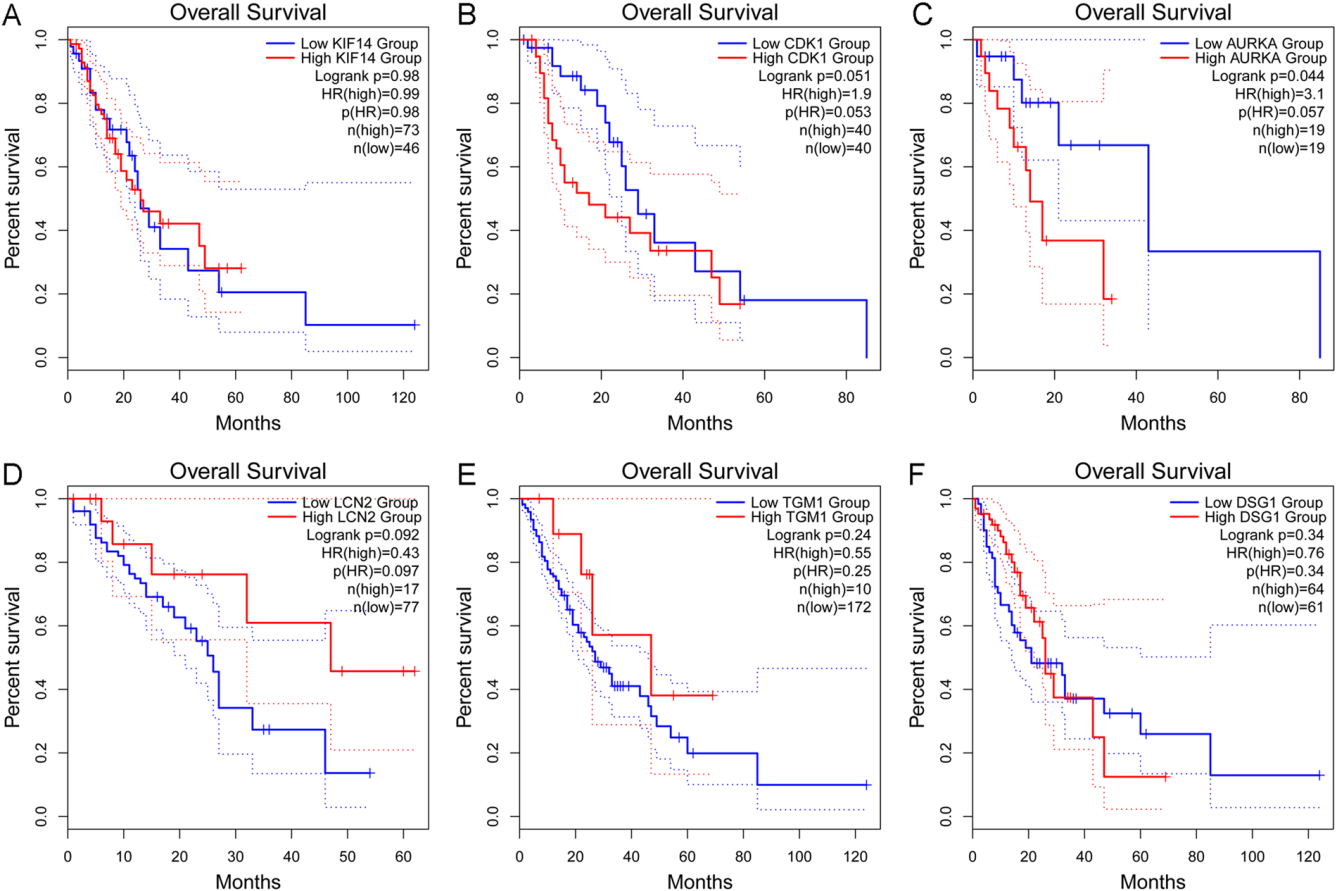
Prognostic value of six hub genes in ESCC. Prognostic value of (**A**) KIF14, (**B**) CDK1, (**C**) AURKA, (**D**) LCN2, (**E**) TGM1, and (**F**) DSG1 were detected in GEPIA database. The survival curve comparing the patients with high (red) and low (blue) expression in ESCC.

According to previous research, the expression of the six hub genes, namely, DSG1, AURKA, CDK1, LCN2, KIF14 and TGM1 were demonstrated in normal esophageal tissue and ESCC. The prognostic value of six hub genes was assessed via the GEPIA database. In addition, we made box plots to visualize the relationships of the expression levels of the 6 key DMGs. Patients with high expression of AURKA were associated with shorter overall survival (Fig. 8). Although p-value is not statistically significant, it was survival significance from the perspective of picture trend that patients with high expression of CDK1 and low expression of LCN2 and TGM1 were associated with shorter overall survival.

## Discussion

Although surgery, chemotherapy and radiation therapy techniques have been greatly improved, the prognosis and survival rate of ESCC are still poor, given the challenges and difficulties in the screening and diagnosis of ESCC. Recently, molecularly targeted therapeutics has been carried out, but they have not exhibited beneficial effects on the long term prognosis of ESCC. Exploring the potential mechanisms of ESCC initiation and development will greatly benefit the diagnosis, treatment and prognosis assessment. In this study, we identified 291 hypomethylated/ high-expression genes and 168 hypermethylated/low-expression genes via multiple bioinformatics tools. Enrichment of these genes demonstrated that aberrant methylation indeed affects certain pathways and hub genes. Our findings can provide novel insight into the explanation of ESCC pathogenesis.

The GO enrichment analysis revealed that the primary biological processes of the hypomethylated/high-expression genes were involved in the regulation of extracellular matrix organization, extracellular structure organization, mitotic cell cycle process, cell cycle process, collagen catabolic process and nuclear division, while the hypermethylated/low-expression genes were primarily linked to response to cAMP, purine-containing compound, oxygen-containing compound, muscle system process, inorganic substance. Previous research has shown that altered expression of particular genes may affect ESCC cell metastasis, invasion, apoptosis and proliferation^15–17^. Our results agree with the fact that carcinoma cell metastasis and invasion are closely associated with abnormal cell adhesion, endodermal cell division and nuclear division^18–20^. Extracellular matrix (ECM), is a critical component of the cancer cell niche, it can provide the tissue with the mechanical support and also mediate the cell-microenvironment interactions^21,22^. It is noteworthy that collagens are one of the major proteins found within the ECM and are associated with many aspects of tumorigenesis^23^. Therefore, this is consistent with the research results that the activation of these cellular processes through ECM is a major cause of tumorigenesis, progression and metastasis. The structure of cytoskeleton is regulated by many cytokines, and thecytoskeleton are closely related to the occurrence and development of tumors^24^. Furthermore, the enriched KEGG pathways of hypomethylated highly expressed genes were mainly linked to ECM-receptor interaction, Focal adhesion, Amoebiasis, PI3K-Akt signaling pathway and Cell cycle. ECM is the first barrier that prevents tumor metastasis^25,26^. Tumor angiogenesis and destruction of the extracellular matrix are two important conditions for tumor invasion and metastasis^27^. It has been reported that the expression of Focal adhesion protein is related to the occurrence, cell differentiation, invasion, lymph node metastasis and prognosis of esophageal cancer and the prognosis of patients with positive expression of Focal adhesion protein is worse than that of patients with negative expression^28^. The study showed that the related protein factors in thePI3K/Akt signaling pathway are highly expressed in esophageal squamous cell carcinoma, and that the pathway is associated with the microvascular formation of esophageal squamous cell carcinoma, and the invasion and metastasis of cancer cells^29^. In contrast, hypermethylated/low-expression genes were related to Pancreatic secretion, Renin secretion, Amphetamine addiction, Salivary secretion and cAMP signaling pathway. Abnormal pancreatic secretion plays an important role in the occurrence and development of reflux esophagitis. Eliminating reflux of pancreatic and bile in Barrett’s esophagus patients can inhibit the progression from atypical hyperplasia to adenocarcinoma^30,31^. Renin can be converted to angiotensin under certain conditions. Studies have found that angiotensin II receptor 1 is related to the differentiation of esophageal squamous cell carcinoma^32^. Angiotensin II and angiotensin II receptor 1 may participate in the occurrence and development of esophageal squamous cell carcinoma, but not via the invasion of esophageal squamous cell carcinoma and lymph node metastasis^33^. As a second messenger in cells, cAMP plays an important role in cell growth, proliferation and differentiation^34^. cAMP is closely related to the occurrence and development of tumors. It has been reported that the growth of tumor cells has a certain relationship with the decrease in intracellular cAMP content^35^. Increasing the content of cAMP can inhibit the growth of tumors and induce the apoptosis of tumors. The content of cAMP was positively correlated with the treatment of tumors. Therefore, cAMP can be used in the treatment and prevention of tumors in the clinic, and has high clinical value^36^.Together, these data suggested that detecting these aberrant signaling pathways could precisely predict tumor progression.

The PPI network of hypomethylated/high-expression genes provides an approach to identifying the functional associations between them and the top three hub genes were selected from this: KIF14, CDK1 and AURKA. The kinesin family member 14 (KIF14) gene, a member of the kinesin superfamily (KIFS), promotes tumorigenesis and progression by regulating the formation of mitotic spindles, chromosome segregation, and cytokinesis^37^. The dysregulation of KIF14 has been confirmed in many human malignancies. Huang *et al*. found that KIF14 was highly expressed in glioma samples and positively correlated with its pathological grade and proliferative activity^38^. Knockdownof KIF14 by siRNA can inhibit the growth of independent anchorage and induce G2/M arrest, which leads to a decrease in the phosphorylation and activity of Akt, thus inducing apoptosis and cytokinesis failure of glioma cell lines and inhibiting the growth of tumors. Osako *et al*. validated that the overexpression of KIF14 in ESCC clinical specimens^39^. It has also been found that downregulation of KIF14 expression can significantly inhibit the proliferation of esophageal squamous cell carcinoma cells and arrest the cell cycle in the G0/G1 phase. In addition, the absence of KIF14 may enhance the sensitivity of tumors to chemotherapy^40^.Therefore, the study of the relationship between KIF14 and cancer and the related mechanism will be a powerful basis for early diagnosis, targeted therapy and prognosis prediction in cancer.

CDK1 (Cyclin-dependent kinase 1) is a serine/threonine protein kinase, that plays a key role in cell proliferation at the G2/M point of the cell cycle. At present, CDK1 is a necessary condition for cell division of all eukaryotic cells. Errors in the regulation mechanism of CDK1 directly lead to cell differentiation disorders, cell cycle disorders, malignant cell proliferation and abnormal transformation, and ultimately malignant tumor formation^41^. Previous studies have found that CDK1 expression was higher in breast cance^42^, oral squamous cell carcinoma^43^, cervical cancer^44^ and gastric cancer^45^ compared to normal tissues, and positively correlated with lymph node metastasis, differentiation, clinical stage and histopathological stage. In esophageal tumors, Expression of CDK1 in ESCC was significantly higher than that in norm esophageal^46^. CDK1 may be a biomarker that could be used for screening tumors, early prognosis and molecular targeted therapy.

Aurora kinases, including Aurora-A, Aurora-B and Aurora-C are a new serine/threonine kinase found in recent years that are involved in mitosis, Aurora-A, or AURKA, regulates the functions of centrosomes and microtubules to ensure the correct separation of centrosomes and the complete division of the cytoplasm^47^. The mutation or overexpression of AURKA may also cause carcinogenesis. AURKA is highly expressed in many tumors, such as breast^48^, ovarian^49^, gastric^50^ and colon cancers^51^. The abnormal expression of AURKA in various solid tumors is closely related to tumor occurrence and development. Importantly, some reports indicate that high expression of AURKA contributes to the development of ESCC^52^. Therefore, exploring the relationship between AURKA and esophageal cancer and its mechanism might help to provide a new target for the treatment of esophageal cancer.

KIF14, CDK1 and AURKA may all be abnormally methylated genes that modulate the cell cycle and proliferation in ESCC. Regarding the hypermethylated/low-expression genes, the most prominent hub genes were LCN2, TGM1 and DSG1.

The gene lipocalin 2 (LCN2) encodes a protein belonging to the lipocalin family. Members of this family transport small hydrophobic molecules such as lipids, steroids and retinols. The protein encoded by this gene is a neutrophil gelatinase-associated lipocalin that acts by injecting iron-containing carriers to limit bacterial growth and plays a role in innate immunity^53^. The presence of this protein in blood and urine is an early biomarker of acute kidney injury^54^. This protein is thought to be involved in a variety of cellular processes, including maintaining the balance of the skin’s internal environment and inhibiting invasion and metastasis. Mice lacking this gene are more susceptible to bacterial infections than wild-type mice. In addition, it was found that the expression of LCN2 was different in tumors, but it was confirmed that LCN2 played a key role in the differentiation, proliferation, angiogenesis, invasion and metastasis of tumors^55^. HPA database analysis revealed thatLCN2 proteins were medium expressed in normal esophageal tissues, while no expression was observed in ESCC tissues. In contrast to our finding, Zhao *et al*. found that endogenous LCN2 expression was increased by activation of the ERK signaling pathway, which promoted the invasion and migration of ESCC^56^.

Transglutaminase 1 (TGM1), the main subtype of three TGM genes expressed in the epidermis and stratified squamous epithelium, encodes for calcium-dependent mercaptase TGM1. TGM1 has two forms in the cytoplasm and membrane junction and has the ability to transfer amino acids to glutamic acid residues in proteins to form isopeptide bonds^57^. Current studies have shown that the mutation of the TGM1 gene is closely related to lamella stratified ichthyosis^58^ and may also affect the proliferation and differentiation of breast cancer cells^59^. Other studies have found that high expression of TGM1 in non-small-cell lung cancer may be conducive to stable adhesion between cancer cells^60^. In addition, researchers found that the mechanism by which TGM1 regulates the development of gastric cancer may be related to the Wnt signaling pathway. When the expression of TGM1 is inhibited, Wnt signaling pathway activity is significantly reduced^61^. Similar to our research, Zhong *et al*. found that the level of TGM1 was increased in ESCC^56^. Recent studies have shown that the expression of transglutaminase 3 (TGM3) in normal esophageal squamous epithelium is significantly reduced than cancer tissue^62^. TGM3, mainly expressed by the superficial epithelial layers of normal head and neck mucosa, is a key factor in the terminal differentiation of keratinocytes. Analysis of the changes and mechanisms of TGM3 in the occurrence and development of ESCC is expected to reveal a molecular marker for the early detection of ESCC.

Desmoglein 1 (DSG1) is a Ca2 + -dependent adhesin desmosomal cadherin that is an important component of desmoglein. DSG1 widely exists in the epithelium, myocardium and other tissues and plays an important role in cell-to-cell junctions. In recent years, DSG1 was been found to have abnormal expression or function in skin, mucosa and tumors. It was found that the expression of Dsg1 in cancer cell lines decreased or even disappeared compared to that in normal control cells^63^. At the same time, Krunicl *et al*. observed by electron microscopy that the number and distribution density of desmosomes on the surface of squamous cell carcinoma cells decreased or even disappeared^64^. These results fully reflect that the decrease in cell adhesion molecules is closely related to the biological malignant behavior of squamous cell carcinoma. Other studies have shown that the expression of DSG1 in the esophageal mucosa of patients with eosinophilic esophagitis is significantly decreased compared to that in normal controls^56^.

## Conclusion

Esophageal squamous cell carcinoma (ESCC) is one of the most common malignancies worldwide, however the molecular mechanism of the occurrence and development of ESCC has not been fully studied.Previous reports have mostly analysed only methylation or only gene expression data and have not been sufficiently powered to discover hub gene expression data, and have not been sufficiently powered to discover hub genes and pathways^65^. Our study combined the analysis of gene expression profiling microarrays and gene methylation profiling microarrays via bioinformatics analysis of available microarray data. In this way, it is possible to develop more precise and reliable screening results. However, we only validated candidate abnormally methylated genes that were differentially expressed using the Oncomine and TCGA databases. Further experiments will be necessary to confirm that these genes and pathways are linked to ESCC.

In conclusion, our research offers a comprehensive bioinformatics analysis of aberrantly methylated DEGs associated with multiple signaling pathways that may be involved in the occurrence and development of ESCC.In addition, the six most modified hub genes were identified, includingKIF14, CDK1, AURKA, LCN2, TGM1, and DSG1.These new discoveries may provide insights for unraveling the pathogenesis of ESCC, and these candidate genes may be optimal abnormal methylation-based biomarkers for the precise diagnosis and treatment of ESCC.Further molecular biological experiments are required to verify the function of the identified candidate genes in ESCC.

## Methods

### Microarray data

The gene expression microarray of GSE20347 and GSE38129, and the gene methylation microarray GSE52826 were downloaded from the Gene Expression Omnibus (GEO, https://www.ncbi.nlm.nih.gov/geo/) of the National Center for Biotechnology Information (NCBI)^66,67^. In total, 17 ESCC and 17 normal specimens were obtained from GSE20347, while 30 ESCC and 30 normal samples were obtained from GSE38129. Both expression microarrays used the platform GPL571: [HG-U133A_2] Affymetrix Human Genome U133A 2.0 Array. For the gene methylation profiling microarray, GSE52826 included a total of 4 ESCC tumour samples and 4 adjacent normal surrounding tissue samples. The platform of this methylation microarray was GPL13534 (Illumina Human Methylation Bead Chip).

### Data acquisition and processing

We used the GEO2R online tool to analyze the raw data of microarrays and identify DMGs and DEGs between the tumor tissues and normal tissues. GEO2R (http://www.ncbi.nlm.nih.gov/geo/geo2r/) is an interactive web toll that allows users to compare different groups of samples in a GEO series to examine differentially expressed genes according to experimental conditions. | log FC| > 1 and P < 0.05 were used as the cut-off standards to obtain DEGs, and | t| > 2 and P  <  0.05 were used as the cut-off standards to find DMGs. Eventually, a Venn diagram was used to identify hypermethylated/low-expression genes and hypomethylated/high-expression genes.

### Functional and pathway enrichment analysis

Gene ontology (GO) analysis and Kyoto Encyclopedia of Genes and Genomes (KEGG) pathway enrichment analysis were conducted on selected genes that were hypomethylated/high-expression and hypermethylated/low-expression via DAVID (DAVID, https://david.ncifcrf.gov/). DAVID is the Database for Annotation, Visualization and Integrated Discovery and is often used to perform functional and pathway enrichment analysis, which offers systematic and integrative functional annotation tools for investigators to unravel biological meaning behind a large list of genes^68^. P < 0.05 was regarded as statistically significant.

### Generation and analysis of a protein–protein interaction (PPI) network

PPI analysis is essential for illustrating the molecular mechanisms of key cellular activities in carcinogenesis. We used the STRING database (https://string-db.org/) to construct a PPI network of hypomethylated/high-expression genes and hypermethylated/low-expression genes^69,70^. An interaction score of 0.4 was regarded as the cut-off criterion and the PPI was visualized. Then, MCODE was conducted to screen modules of the PPI network with MCODE score>4 and number of nodes >4 in Cytoscape software. The top 3 hub genes were selected by the CytoHubba app in Cytoscape software. To determine the expression pattern of six hub genes in ESCC, the datasets were analyzed in Oncomine (https://www.oncomine.org). Oncomine is an online database consisting of previously published and open-access microarray data^71^. The analysis enables multiple comparisons of gene expression between different studies, the importance of the gene expression across the available studies was also taken into account. The consequences were filtered by selecting ESCC vs. normal tissue.

### Hub gene validation in TCGA database

The Cancer Genome Atlas (TCGA) database, collaboration between the National Cancer Institute and National Human Genome Research Institute, has generated comprehensive, multi-dimensional maps of the key genomic changes in various types of cancers^72^. MEXPRESS (http://mexpress.be/) is a data visualization tool designed for the easy visualization of TCGA expression^73^, DNA methylation and clinical data, as well as the relationships between them. In TCGA set validation, the top 20 genes that with high DMGs were selected. To confirm the results, we used the MEXPRESS to validate hypermethylated/low-expression hub genes and hypomethylated/ high-expression hub genes in the TCGA database. The human Protein Atlas is a website that involves immunohistochemistry-base expression data for distribution and expression of 20 tumor tissues, 47 cell lines, 48 human normal tissues and 12 blood cells. In our study, direct contrast of protein expression of different hub genes between normal and HNSCC tissues was used by immunohistochemistry image. The probability of survival and significance were calculated using the GEPIA database. GEPIA is a newly created online interactive web server which enables users to explore the RNA sequencing expression information of tumors/normal tissues or samples from the Genotype Tissue Expression (GTEx) projects and The Cancer Genome Atlas (TCGA), based on a criterion processing pipeline. GEPIA offer customizable functions such as profiling regarding to pathological stages, cancer types, differential expression analysis, survival analysis, correlation analysis and similar gene detection.

## Supplementary information

Supplementary information.

Supplementary material 2.

## Data Availability

The datasets generated and/or analysed during the current study are available in the GEO repository, https://www.ncbi.nlm.nih.gov/geo/.
