# Supplementary material for: Identification of candidate aberrantly methylated and differentially expressed genes in Esophageal squamous cell carcinoma
Source: Sci Rep. 2020 Jun 16;10:9735. doi: 10.1038/s41598-020-66847-4 (PMC7297810; doi:10.1038/s41598-020-66847-4)
Supplement: Supplementary file 1 — Supplementary information. [file 41598_2020_66847_MOESM1_ESM.docx]

**Identification of candidate aberrantly methylated and differentially expressed genes in Esophageal squamous cell carcinoma**

Bao-Ai Han^1*^, Xiu-Ping Yang^4*^, Davood K Hosseini^3,5^, Bo Zhang^6^, Ya Zhang^4^, Jin-Tao Yu^2^, Shan Chen^2^, Fan Zhang^7^, Tao Zhou^2^, Hai-Ying Sun^2,3^

^1^Public Laboratory, Key Laboratory of Breast Cancer Prevention and Therapy, Ministry of Education, Tianjin Medical University Cancer Institute and Hospital, National Clinical Research Center for Cancer, Tianjin Medical University, Tianjin 30000, China.

^2^Department of Otorhinolaryngology, Union Hospital, Tongji Medical College, Huazhong University of Science and Technology, Wuhan 430022, China.

^3^Department of Otolaryngology-Head and Neck Surgery, Stanford University School of Medicine, Stanford 94305，United States of American

^4^Department of Otorhinolaryngology, Head and Neck Surgery, Zhongnan Hospital of Wuhan University, Wuhan, 430071, China.

^5^Department of Medicine, Stanford University School of Medicine, Stanford 94305，United States of American

^6^Department of Neurosurgery, Tongji Hospital, Tongji Medical College, Huazhong University of Science and Technology, Wuhan 430030, China.

^7^Guangdong Provincial Maternal and Child Health Care Hospital, Guangzhou 511400, China.

^*^ Contributed equally to this article.

*Correspondence to:*

Tao Zhou, Department of Otorhinolaryngology, Union Hospital, Tongji Medical College, Huazhong University of Science and Technology, Wuhan, 430022,P.R. China.

E-mail: [entzt2013@sina.cn](mailto:entzt2013@sina.cn)

Haiying Sun, Department of Otorhinolaryngology, Union Hospital, Tongji Medical College, Huazhong University of Science and Technology, Wuhan, 430022, P.R. China.

Department of Otolaryngology-Head and Neck Surgery, Stanford University School of Medicine, Stanford, 94305, United States of American

E-mail: [momo426@stanford.edu](mailto:momo426@stanford.edu)

**S1:** P Value of the gene ontology annotation and pathway enrichment analysis of all the aberrantly methylated and differentially expressed genes.
